# A novel diagnostic model combining ProGRP and inflammatory biomarkers for early detection of lung cancer: development and validation in treatment-naive cohorts

**DOI:** 10.1186/s12885-026-15961-z

**Published:** 2026-04-06

**Authors:** Jin Ma, Shumin Zhu, Xinyuan Li, Shichao Gao, Yulan Geng

**Affiliations:** 1 Department of Laboratory Medicine, The Third Hospital of Hebei Medical University, 139 Ziqiang Road, Shijiazhuang, 050000 China; 2 Department of Laboratory Medicine, The First Hospital of Hebei Medical University, 89 Donggang Road, Shijiazhuang, 050031 China; 3 Department of Laboratory Medicine, Hebei Children’s Hospital, 133 Jianhuan Road, Shijiazhuang, 050031 China

**Keywords:** Lung cancer, Early diagnosis, Biomarker, Inflammation

## Abstract

**Background:**

Lung cancer remains the leading cause of cancer-related mortality globally, with late-stage diagnosis contributing to poor outcomes. Non-invasive biomarkers capable of complementing existing screening modalities are urgently needed to improve early detection.

**Methods:**

This retrospective study developed and validated a diagnostic model using serum tumor markers and inflammation-related biomarkers. The training cohort included 110 treatment-naive lung cancer patients and 107 healthy controls, while an external validation cohort comprised 44 lung cancer patients and 52 controls. Compare the differences in tumor markers, cytokines, and indices derived from whole blood cell counts between the lung cancer group and the healthy control group. Logistic regression analysis was used to identify independent risk factors for lung cancer and to construct a clinical prediction model. The model’s discrimination, calibration, and clinical applicability were assessed using receiver operating characteristic (ROC) curves, the Hosmer-Lemeshow test, and decision curve analysis (DCA), among other methods.

**Results:**

The predictive model demonstrated high diagnostic accuracy in the training cohort, with an AUC of 0.972 (sensitivity 89.2%, specificity 99.1%). External validation showed an AUC of 0.897 (sensitivity 72.7%, specificity 98.1%), confirming its good generalizability. Notably, Pro-Gastrin Releasing Peptide(ProGRP)and inflammatory markers were independently associated with the risk of lung cancer (*P* < 0.05). DCA analysis indicated that the model has significant clinical utility across a wide range of probability thresholds (0–80%).

**Conclusion:**

This study presents a high-performance, non-invasive diagnostic model integrating ProGRP and systemic inflammatory biomarkers for lung cancer detection. The combined model outperforms individual biomarkers and may enhance early lung cancer screening, particularly in settings where imaging modalities are limited. Further multi-center prospective studies are warranted to validate its clinical applicability.

## Introduction

Lung cancer persists as a leading global health challenge, accounting for the highest incidence and mortality among malignancies. In 2022, an estimated 2.48 million new lung cancer cases and 1.8 million deaths were reported worldwide, with projections suggesting a dramatic rise to 4.62 million cases and 3.55 million deaths by 2050 [1]. Notably, Asia shoulders the greatest burden, contributing to 60% of new diagnoses and 62% of lung cancer-related fatalities exceeding rates in Europe and the United States [2]. Although China’s overall cancer incidence remains lower than that of Western nations, the lung cancer mortality and disability-adjusted life year (DALY) burden are escalating rapidly, underscoring an urgent need for improved early detection strategies [3]. Early diagnosis is pivotal for enhancing therapeutic outcomes and reducing mortality [4, 5]. However, current diagnostic approaches primarily imaging and invasive biopsies are limited by suboptimal sensitivity for early-stage disease, high false-positive rates, and risks associated with repeated radiation exposure [6–9]. While low-dose computed tomography (LDCT) screening has reduced lung cancer mortality in high-risk populations, its implementation is hindered by cost, accessibility, and variable adherence [10]. Consequently, non-invasive biomarkers capable of complementing imaging modalities are critically needed to refine risk stratification and early detection [11–13].

Emerging evidence highlights the interplay between systemic inflammation and lung cancer pathogenesis, mediated through chronic immune activation, oxidative stress, and tumor microenvironment remodeling [14]. Inflammatory indices derived from complete blood counts (CBC) such as the systemic immune-inflammation index (SII), neutrophil-to-lymphocyte ratio (NLR), and platelet-to-lymphocyte ratio (PLR) have shown prognostic value in lung cancer [15, 16]. Concurrently, serum biomarkers like cytokeratin fragment 19 (CYFRA21-1), neuron-specific enolase (NSE), and ProGRP are routinely used in clinical practice, though their diagnostic accuracy for early-stage disease remains modest [17]. Recent studies further implicated macrophage-associated cytokines Interleukin-6(IL-6)、Interleukin-12p70༈IL-12p70༉in lung cancer progression, suggesting their potential as auxiliary diagnostic markers [18].

Despite these advances, integrated models combining tumor markers, inflammatory indices, and cytokines for lung cancer diagnosis are underexplored, particularly in treatment-naïve early-stage cohorts. Here, we evaluated the diagnostic utility of complete blood cell (CBC)-derived indices, serum biomarkers, and cytokines in lung cancer patients versus healthy controls. Using logistic regression, we developed a diagnostic model and validated its performance in an independent cohort. Our findings provide a robust, non-invasive tool to enhance early lung cancer detection and inform risk-based screening protocols.

## Materials and methods

### Study subjects

A total of 133 hospitalized patients diagnosed with lung cancer and 142 healthy controls were initially enrolled from January, 2021 to September, 2024.The healthy control group consisted of individuals who underwent health check-ups at our hospital. Those confirmed to be healthy through physical examination, medical history review, and imaging studies were included, while individuals with malignant tumors or chronic inflammatory diseases were excluded.After excluding cases with incomplete baseline data or failure to meet inclusion criteria, the final analysis included 110 lung cancer subjects and 107 healthy controls. Among the 110 lung cancer patients, pathological subtype information was available for 76 patients (69.1%). The distribution was as follows: adenocarcinoma (*n* = 51, 67.1%), squamous cell carcinoma (*n* = 15, 19.7%), and small cell lung cancer (*n* = 10, 13.2%). This distribution is consistent with global epidemiological patterns, where non-small cell lung cancer (NSCLC) accounts for approximately 85% of all lung cancers. Notably, the proportion of SCLC (13.2%) was lower than the typical 15–20% reported in literature, suggesting that the diagnostic performance of our model is not inflated by SCLC cases.Additionally, another 96 individuals(44 lung cancer patients and 52 healthy controls)from the same hospital as an external validation cohort were enrolled in this study from November, 2024 to March, 2025.

**Fig. 1:**
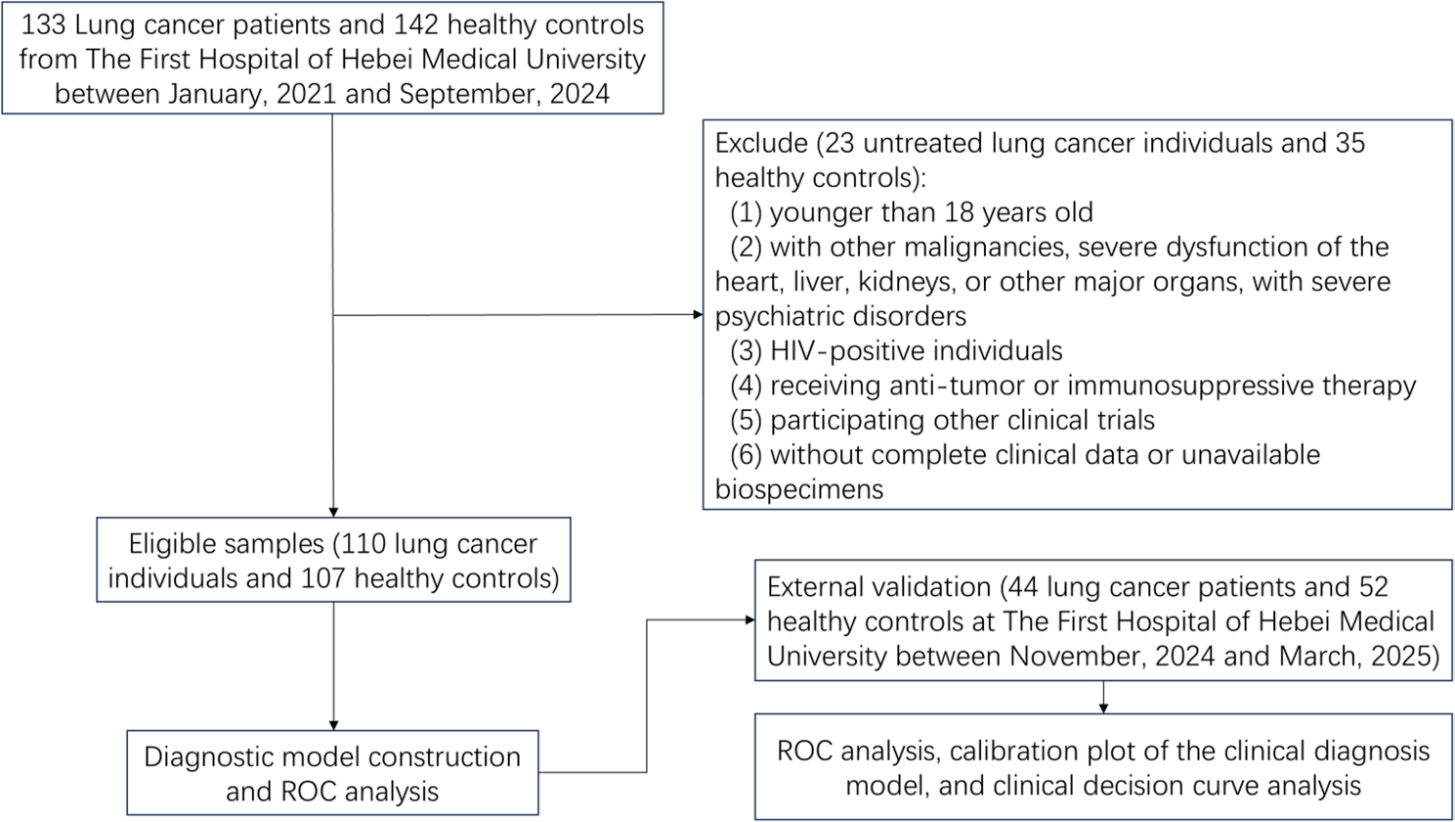
Flowchart of the study

All participants were aged 18 or older. The lung cancer patients were diagnosed through histopathology or cytology and only were in the treatment-naïve early-stage.Early-stage patients are those who are admitted to the hospital due to abnormal pulmonary imaging findings, and the initial diagnosis of lung cancer is confirmed by pathological examination upon admission. Complete baseline data were available, including sex, age, pathological diagnosis, for all participants. Patients were excluded if they (i) were younger than 18 years, (ii) with other malignancies, severe dysfunction of the heart, liver, kidneys, or other major organs, with severe psychiatric disorders, (iii) HIV-positive individuals, (iv) receiving immunosuppressive therapy, (v) participating other clinical trials, (vi) without complete clinical data or whose biospecimens were unavailable. The flowchart of the present study showed as Fig. 1.

### Laboratory measurements of CBC, tumor-related markers, and cytokines

All blood samples were collected in the morning after an overnight fast (8–12 h) to minimize the influence of dietary status and diurnal variation on biomarker levels, particularly inflammatory markers like IL-6.

Tumor markers are analyzed using the MAGLU MI 8 fully automated chemiluminescence immunoassay analyzer from Shenzhen New Industry. All reagents used are original matched reagents. By collecting serum samples from fasting patients, detection is carried out using electrochemiluminescence technology, including CYFRA21-1, NSE, Squamous Cell Carcinoma Antigen(SCCA), and ProGRP.

Cytokine detection was performed using the Beckman Navious flow cytometer, with reagents from Jiangxi Saiji Biotechnology Co., Ltd. Peripheral blood samples were collected, and specific antibodies were used to label cells with fluorophores or enzymes. The flow cytometer then excited the fluorophores and detected fluorescence signals at different wavelengths to quantify cytokine expression levels, including Interleukin-1 beta(IL-1β), IL-6, Tumor Necrosis Factor-alpha༈TNF-α༉, Interferon-gamma༈IFN-γ༉, and IL-12p70.

The complete blood count uses the Beckman DXH800 blood routine analyzer, and the reagents used are the original supporting reagents of the supporting instruments, and the patient’s peripheral blood samples are collected and EDTA is used as an anticoagulant, and the samples are put into the DXH800 blood routine analyzer for testing. The instrument uses multi-angle scattered light analysis technology and new algorithm rules to automatically complete data processing and output counting and classification results including red blood cells, white blood cells, platelets, etc.

### Calculation of CBC-derived inflammatory indices

There are four CBC-derived inflammatory biomarkers, including Systemic Immune-Inflammation Index (SII), Neutrophil-to-Lymphocyte Ratio (NLR), Platelet-to-Lymphocyte Ratio (PLR), and Lymphocyte-to-Monocyte Ratio (LMR). The followings are the calculation formulas:

$$\begin{aligned} \mathrm{SII}=&\:\text{Platelet count}\left(\mathrm{PLT}\right)\times\text{Neutrophil count}\left(\mathrm{NE}\right)\\&/\text{Lymphocyte count}\left(\mathrm{LY}\right) \end{aligned}$$

$$\mathrm{NLR}=\mathrm{NE}/\mathrm{LY}$$

$$\mathrm{PLR}=\mathrm{PLT}/\mathrm{LY}$$

$$\mathrm{LMR}=\mathrm{LY}/\mathrm{MO}$$

### Statistical analysis

All analyses were conducted using the Free Statistics software (v1.9) and R statistical packages. Continuous variables following a normal distribution were expressed as mean ± standard error, while non-normally distributed variables were presented as median (interquartile range). Categorical variables were reported as numbers (percentages).

Comparisons between groups were conducted using the Student’s *t*-test for normally distributed continuous variables and the Mann-Whitney *U* test for non-normally distributed variables. For categorical variables, the chi-square test or Fisher’s exact test was applied, as appropriate.

Binary logistic regression models were employed to determine the adjusted odds ratios (OR) and 95% confidence intervals (CI) for the associations between tumor markers, cytokines, complete blood cell counts, and lung cancer. Multivariable logistic regression analysis was performed to identify independent predictors of lung cancer. A stepwise logistic regression approach was used to construct a diagnostic predictive model, and receiver operating characteristic (ROC) curve analysis was conducted to evaluate the performance of relevant indicators and the diagnostic model. Additional, clinical decision curve analysis (DCA) was used to evaluate the performance of the diagnostic models in the external dataset. A two-sided *P*-value < 0.05 was considered statistically significant.

## Results

### Analysis of clinical baseline data

A comparative analysis of baseline clinical data was performed between 110 lung cancer patients and 107 healthy controls **(**Table 1**)**. The lung cancer cohort comprised 36 females and 74 males, with no significant difference in gender distribution compared to the control group (*P* > 0.05). However, the age distribution between the two groups differed significantly (*P* < 0.05).

**Table 1:** Comparison of baseline characteristics between lung cancer group and healthy group

Clinical variables	Total (*n* = 217)	Healthy group (*n* = 107)	Lung cancer group (*n* = 110)	*P*
Sex, n (%)				0.390
Male	140 (64.5)	66 (61.7)	74 (67.3)	
Female	77 (35.5)	41 (38.3)	36 (32.7)	
Age (years)	58.0 (49.0, 66.0)	51.0 (40.5, 56.0)	66.0 (59.0, 72.0)	< 0.001
CYFRA21-1 (ng/ml)	1.2 (0.6, 2.1)	0.9 (0.2, 1.5)	1.6 (0.9, 4.2)	< 0.001
NSE (ng/ml)	3.5 (2.7, 4.5)	3.0 (2.3, 3.6)	4.3 (3.4, 6.9)	< 0.001
SCCA (ng/ml)	1.6 (1.2, 2.1)	1.4 (1.1, 1.7)	1.7 (1.3, 3.0)	< 0.001
ProGRP (pg/ml)	33.4 (29.3, 44.2)	31.0 (28.6, 33.5)	46.3 (32.8, 70.7)	< 0.001
IL-1β (pg/ml)	1.8 (1.0, 3.0)	1.6 (0.9, 2.5)	1.9 (1.2, 3.3)	0.032
IL-6 (pg/ml)	4.9 (2.9, 10.3)	3.1 (2.2, 4.5)	10.2 (5.4, 31.7)	< 0.001
TNF-α (pg/ml)	2.2 (1.5, 3.1)	2.1 (1.3, 2.9)	2.3 (1.6, 3.5)	0.025
IFN-γ (pg/ml)	1.7 (1.2, 2.6)	1.6 (1.1, 2.5)	1.8 (1.3, 2.6)	0.103
IL-12p70 (pg/ml)	1.7 (1.0, 2.6)	1.7 (0.9, 2.3)	1.9 (1.1, 3.1)	0.008
SII	468.8(355.9, 871.1)	405.3 (311.8, 518.3)	732.9 (425.2, 1392.8)	< 0.001
NLR	2.3 (1.6, 4.3)	1.7 (1.4, 2.2)	4.1 (2.4, 7.0)	< 0.001
PLR	143.8 (112.6, 210.0)	121.1 (101.8, 154.8)	203.2 (129.2, 259.6)	< 0.001
LMR	3.5 (2.0, 5.2)	5.0 (4.0, 6.0)	2.0 (1.3, 3.0)	< 0.001

The two groups showed significant differences in CYFRA21-1, NSE, SCCA, ProGRP, IL-1β, IL-6, IL-12p70, TNF-α, SII, NLR, PLR, and LMR (*P* < 0.05).

### Identification of predictive factors for lung cancer

To identify risk factors associated with lung cancer development, binary logistic regression was performed, with age differences between the two groups adjusted for in the analysis. After age adjustment, significant differences (*P* < 0.05) remained between the lung cancer group and the healthy control group for the following biomarkers: CYFRA21-1, NSE, SCCA, ProGRP, interleukin-6 (IL-6), interleukin-12P70 (IL-12p70), SII, NLR, PLR, and LMR (Table 2). These results indicate that CYFRA21-1, NSE, SCCA, ProGRP, IL-6, IL-12p70, SII, NLR, PLR, and LMR are independent factors influencing lung cancer pathogenesis.

**Table 2:** Lung cancer patients’ cytokines, tumor markers, and lung cancerulated inflammatory indexes logistic regression analysis regression

Items	OR (95%CI)^A^	*P* ^A^	OR (95%CI)^B^	*P* ^B^
CYFRA21-1 (ng/ml)	1.88 (1.42–2.48)	< 0.001	1.96 (1.38–2.79)	< 0.001
NSE (ng/ml)	1.36 (1.15–1.60)	< 0.001	1.26 (1.10–1.44)	0.001
SCCA (ng/ml)	2.09 (1.40–3.13)	< 0.001	2.12 (1.29–3.47)	0.003
ProGRP (pg/ml)	1.15 (1.09–1.21)	< 0.001	1.13 (1.06–1.2)	< 0.001
IL-1β (pg/ml)	1.09 (0.99–1.20)	0.088	1.10 (0.93–1.29)	0.265
IL-6 (pg/ml)	1.63 (1.38–1.92)	< 0.001	1.63 (1.31–2.02)	< 0.001
TNF-α (pg/ml)	1.34 (1.11–1.62)	0.002	1.25 (0.99–1.58)	0.056
IL-12p70 (pg/ml)	1.51 (1.20–1.90)	< 0.001	1.37 (1.04–1.81)	0.026
SII	1.00 (1.00–1.00)	< 0.001	1.00 (1.00–1.00)	0.001
NLR	2.59 (1.89–3.56)	< 0.001	2.38 (1.66–3.41)	< 0.001
PLR	1.01 (1.01–1.02)	< 0.001	1.01 (1.01–1.02)	< 0.001
LMR	0.30 (0.22–0.41)	< 0.001	0.32 (0.23–0.45)	< 0.001

Model A represents the results without any adjustments, and model B represents the results for all biomarkers after adjusting for age

### Construction of predictive model and analysis of ROC curve

After performing stepwise logistic regression on hematological parameters with independent influences on lung cancer, five continuous variables (ProGRP, IL-6, IL-12p70, SII, and NLR), identified as potential risk factors for lung cancer, were further evaluated using ROC curves. The ProGRP, IL-6, IL-12p70, SII, and NLR were all significant predictors for lung cancer.

The logistic regression model was constructed with ProGRP, IL-6, IL-12p70, SII, and NLR. The formula of model was:

$$\begin{aligned} \mathrm{Model}=&-12.947+0.216\times\mathrm{ProGRP}+0.478\times\mathrm{IL}-6+0.325\\&\times\mathrm{IL}-12\mathrm{p}70-0.003\times\mathrm{SII}+1.445\times\mathrm{NLR} \end{aligned}$$

**Fig. 2:**
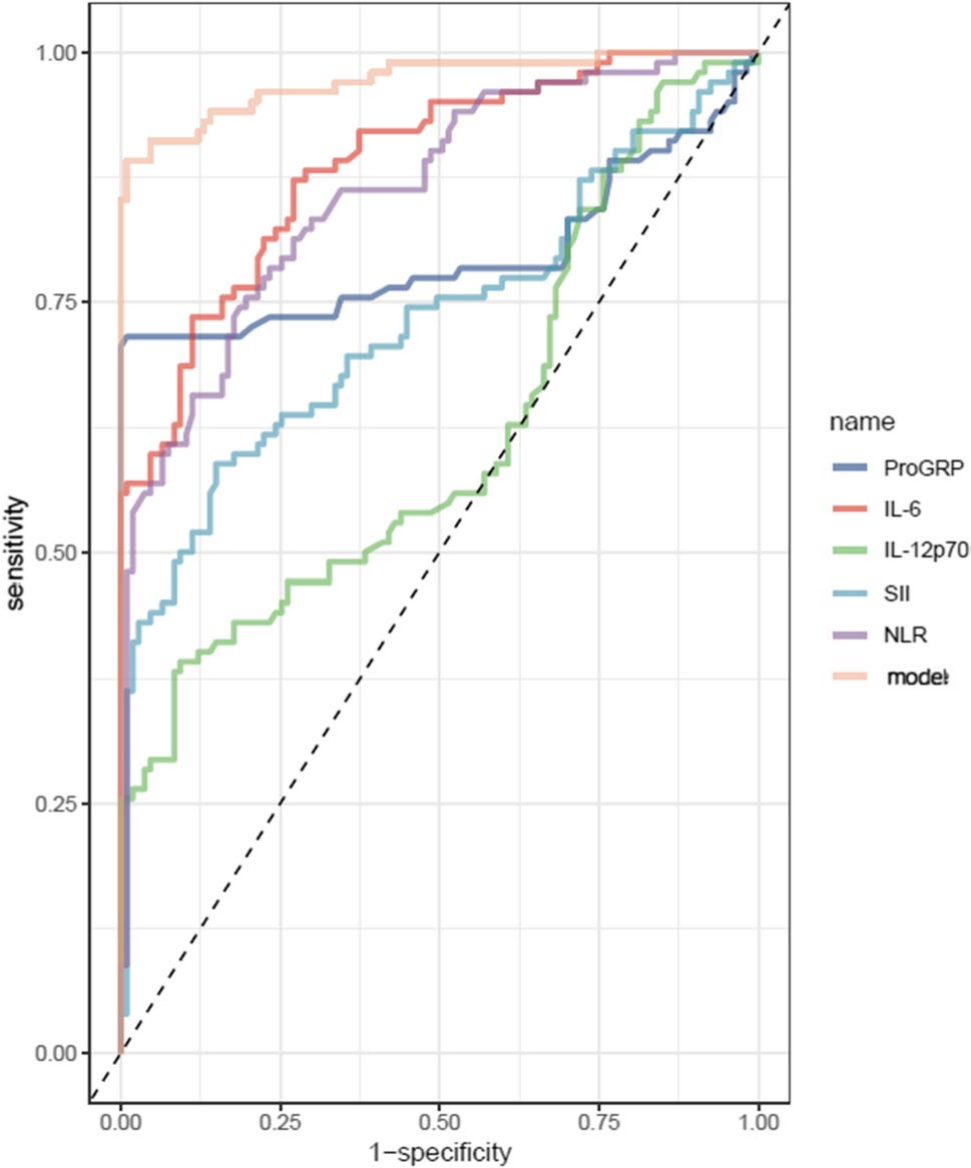
ROC curves for diagnostic indicators

**Fig. 3:**
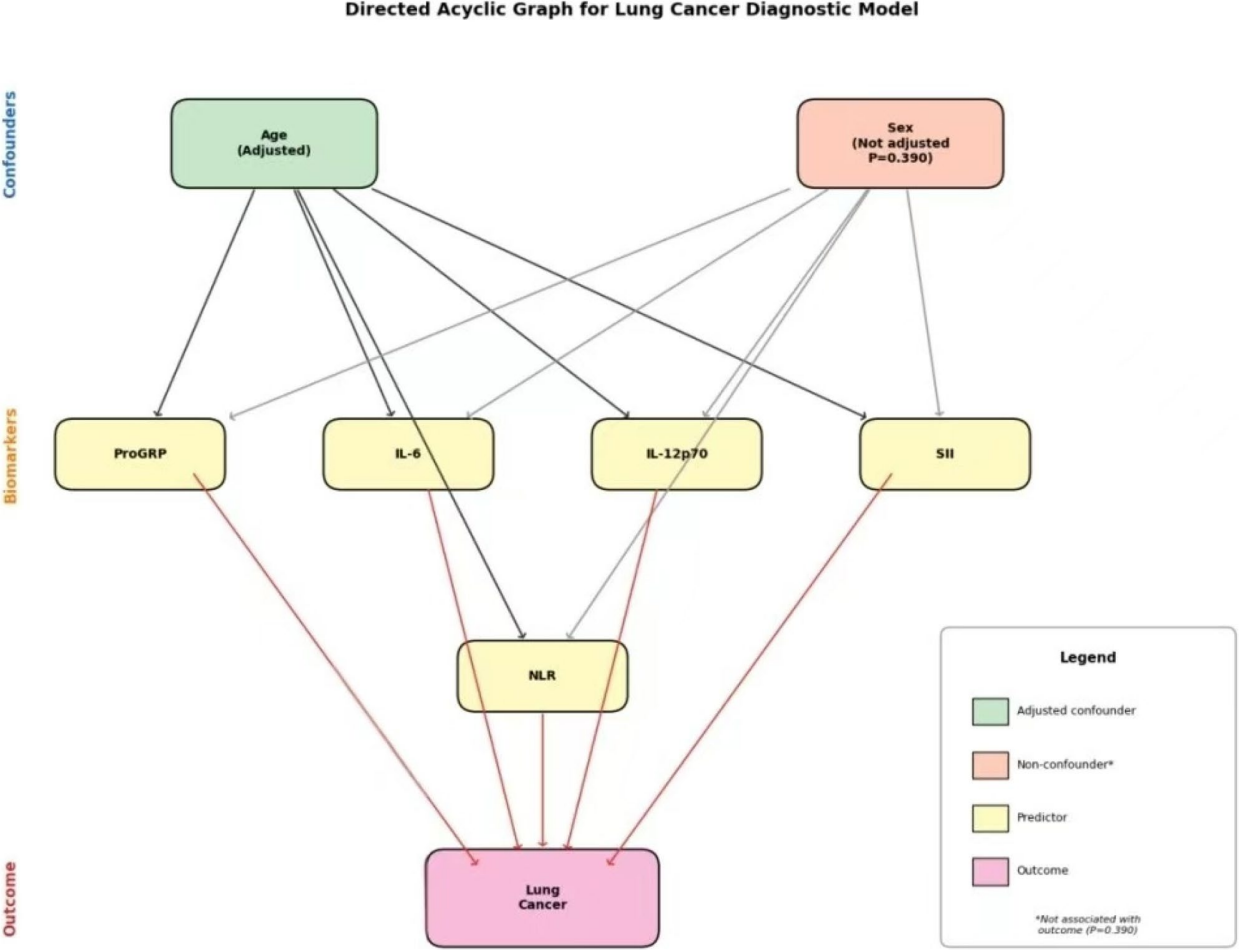
Directed Acyclic Graph (DAG) depicting the relationships between exposure variables, confounders, and lung cancer outcome

The model achieved an AUC of 0.972, with a specificity of 99.1% and a sensitivity of 89.2%, as shown in Table 3 and Fig. 2. Directed Acyclic Graph (DAG) analysis was performed to identify confounders (Fig. 3). Age was identified as the only confounder requiring adjustment; sex was not a confounder (*P* = 0.390 for association with outcome).

**Table 3:** ROC curve analysis for the diagnosis of lung cancer using ProGRP, IL-6, IL-12p70, SII, and NLR

Items	AUC	95%CI	Cut-off	specificity	sensitivity
ProGRP	0.796	0.727 ~ 0.865	35.500	0.991	0.715
IL-6	0.889	0.846 ~ 0.932	5.625	0.888	0.735
IL-12p70	0.621	0.543 ~ 0.698	2.670	0.907	0.392
SII	0.730	0.659 ~ 0.800	630.793	0.851	0.588
NLR	0.859	0.810 ~ 0.909	2.342	0.804	0.755
Model	0.972	0.951 ~ 0.993	0.655	0.991	0.892

### External validation of the model

**Fig. 4:**
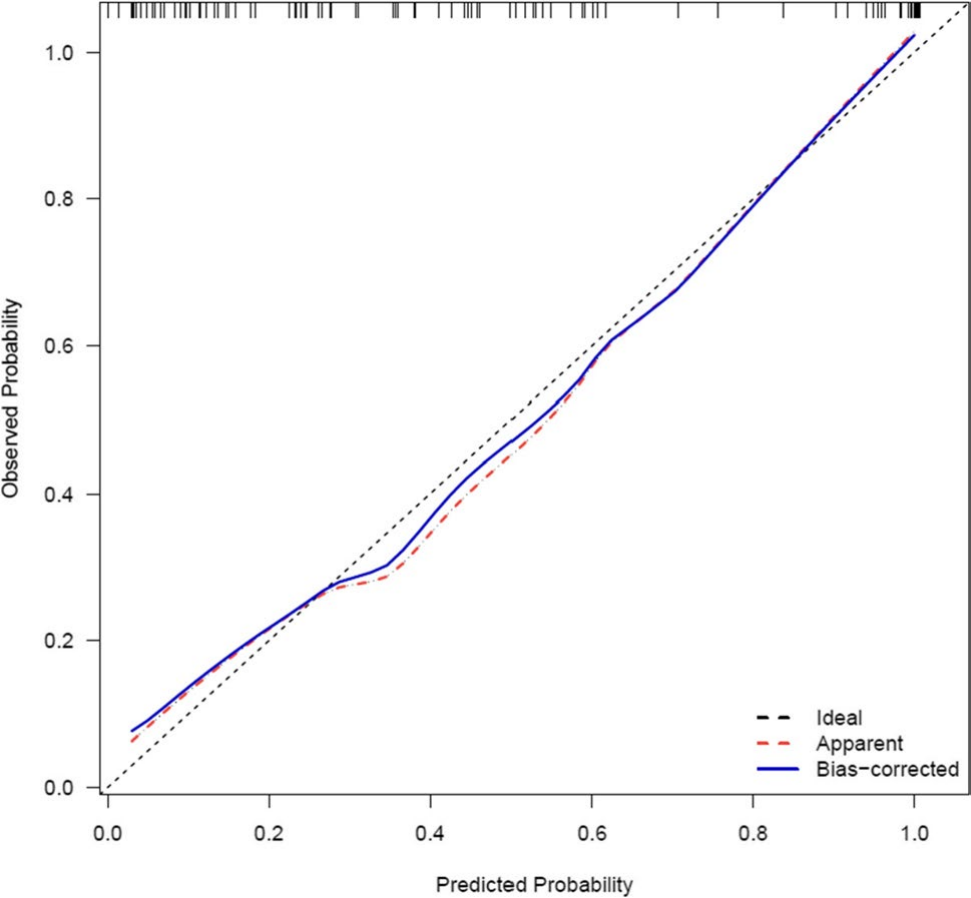
Calibration curve of the clinical prediction model for lung cancer diagnosis

**Fig. 5:**
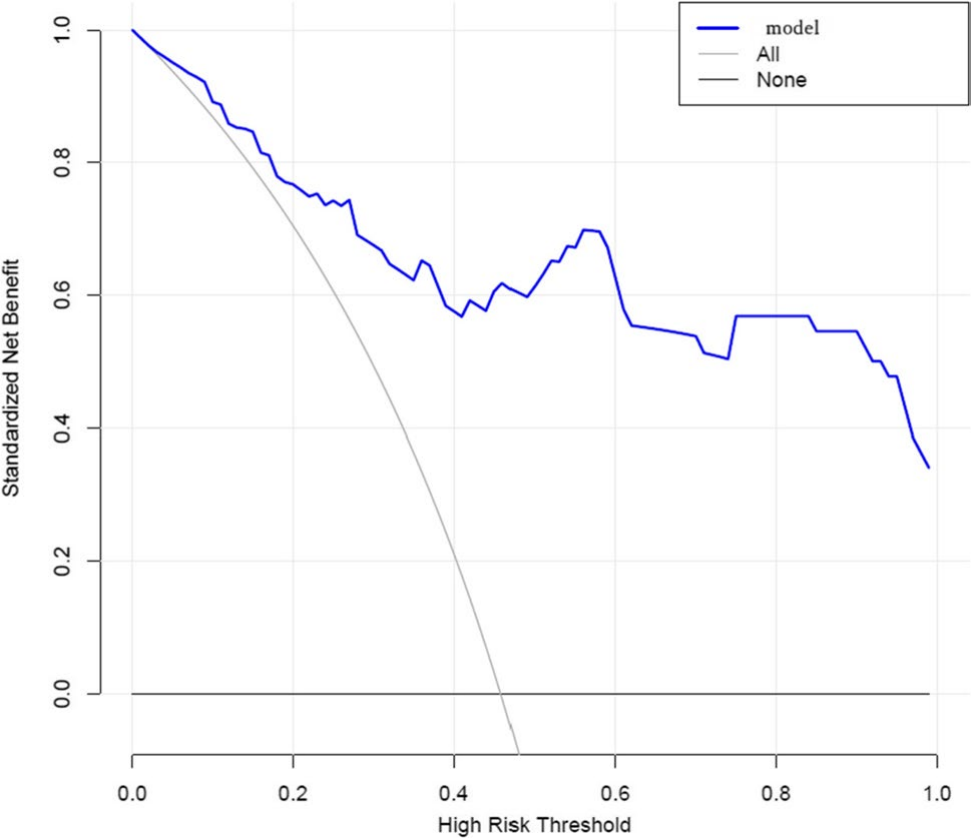
Decision Curve Analysis (DCA) of the clinical prediction model for lung cancer diagnosis

**Fig. 6:**
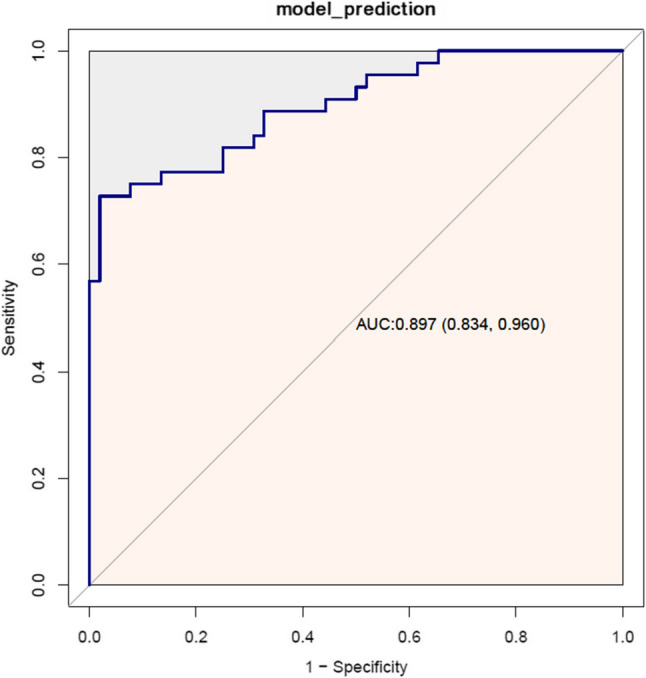
External validation ROC curve analysis of the diagnostic model

The calibration curve (age-adjusted) had a good performance (Hosmer-Lemeshow test: X-squared = 4.833, *P* = 0.775) (Fig. 4), and the clinical decision curve analysis (DCA) indicated that patients achieved a high net benefit for the predicted probability thresholds between 0 and 80% (Fig. 5). External validation was performed using a dataset of 44 lung cancer patients and 52 healthy controls at The First Hospital of Hebei Medical University. Despite the differences in patient characteristics between the two groups, the ROC maintained a good predictive accuracy and discrimination ability with an AUC (C- statistic, C-index) of 0.897 (95% CI, 0.834–0.960),cut-off value of 0.568, specificity of 0.981, and sensitivity of 0.727 (Fig. 6).

## Discussion

This study developed and validated a diagnostic model incorporating serum tumor markers (ProGRP) and inflammation-related biomarkers (IL-6, IL-12p70, SII, and NLR) to distinguish lung cancer patients from healthy controls. Our findings demonstrate that the combined model exhibits high diagnostic accuracy (AUC = 0.972 in the training cohort and 0.897 in the validation cohort), outperforming individual biomarkers. These results align with emerging evidence supporting the integration of inflammatory indices and tumor markers for lung cancer risk stratification and early detection [19].

It is noteworthy that the lung cancer cohort was significantly older than healthy controls (66.0 vs. 51.0 years, *P* < 0.001), reflecting the established epidemiology of lung cancer where incidence rises markedly after age 60. This age difference was anticipated given that lung cancer predominantly affects older adults, while healthy screening populations are typically younger. Importantly, our multivariable model adjusted for age as a covariate, and the consistency of biomarker effects before and after adjustment (Table 2) suggests that age-related variations in inflammatory markers do not substantially confound our findings. The high diagnostic accuracy persisted after age adjustment (AUC = 0.972), indicating that the model captures genuine disease-related signals beyond age-associated biomarker elevation.Directed Acyclic Graph (DAG) analysis was performed to identify potential confounders between exposure variables and lung cancer outcome. Age was identified as the only confounder requiring adjustment in our analysis, while sex was not considered a confounder given its lack of association with the outcome (*P* = 0.390). This approach ensured that our multivariable model appropriately accounted for the primary demographic factor influencing both biomarker levels and disease risk.

The observed elevation in ProGRP, a well-established biomarker for small-cell lung cancer (SCLC) [20], corroborates prior studies that demonstrated its diagnostic utility for neuroendocrine tumors [21, 22]. The histological subtype distribution in our cohort aligns with global epidemiology, with NSCLC comprising 86.8% of cases. Importantly, the SCLC proportion (13.2%) was even lower than the typical 15–20% reported in population-based studies. This indicates that the high diagnostic accuracy of our model (AUC = 0.972) primarily reflects its performance in NSCLC—the predominant form of lung cancer—rather than being driven by SCLC cases where ProGRP is known to have high diagnostic value. Therefore, our findings are generalizable to the broader lung cancer population and do not overestimate diagnostic efficacy for NSCLC.Interestingly.Our model also highlights the significance of IL-6 and IL-12p70, cytokines polarization and chronic inflammation which are known drivers of lung cancer pathogenesis [18, 19, 23]. The positive correlation between systemic immune-inflammation indices (SII, NLR, PLR) and lung cancer risk aligns with several previous reports [24–26]. Our model’s strong performance in external validation (AUC = 0.897) underscores its generalizability, though the slight reduction in sensitivity (72.7%) compared with the training cohort (89.2%) may reflect variations in patient demographics or biomarker measurement protocols. The high specificity (> 98%) minimizes false positives, a critical advantage given that current lung cancer screening modalities such as LDCT suffer from high false-positive rates [27]. Decision curve analysis further confirmed the clinical utility of our model, demonstrating net benefit across a wide probability threshold range (0–80%).

We defined our study population as “treatment-naïve early-stage” lung cancer patients, emphasizing the diagnostic context rather than formal TNM staging. These patients represent a clinically critical population: individuals presenting with abnormal imaging who receive first-time pathological confirmation without prior treatment. This definition aligns with real-world screening scenarios, where the goal is to identify lung cancer at the first diagnostic opportunity—regardless of exact TNM stage—when curative-intent treatment is still feasible.

The absence of uniform TNM staging reflects the retrospective nature of the study and routine clinical practice at our institution, where not all patients underwent complete staging workup (brain MRI, PET-CT) at admission. However, the high proportion of pathologically confirmed cases with available subtype data (adenocarcinoma 67.1%, squamous cell carcinoma 19.7%) suggests a representative distribution of operable lung cancers. The model’s strong performance in this pragmatically defined early population (AUC = 0.972) supports its utility in the intended use case: assisting diagnosis at first presentation, when treatment decisions are being made.

We acknowledge that direct comparison with established diagnostic tools (e.g., low-dose CT, PET-CT, tissue biopsy) was not performed in this study. However, such head-to-head comparison is methodologically problematic for several reasons. First, serum biomarkers and imaging/biopsy address different clinical questions: biomarkers provide biological risk assessment for screening and triage, whereas imaging and histology offer anatomical and definitive diagnosis. Second, these tools target different populations and clinical scenarios.Our model is designed for resource-limited settings or initial risk stratification, while LDCT and invasive procedures are typically employed in specialized centers for definitive diagnosis. Third, reference standard bias would invalidate any direct comparison, as tissue biopsy (the gold standard for diagnosis) is fundamentally different from serum biomarkers in both nature and clinical application.Rather than replacing existing diagnostics, our biomarker model is intended to complement them by identifying high-risk individuals who may benefit from further imaging workup, thereby reducing unnecessary invasive procedures and healthcare costs. This complementary positioning aligns with emerging strategies in precision screening, where multi-modal approaches integrating biomarkers and imaging are advocated to optimize diagnostic efficiency.

We acknowledge that smoking status and tobacco consumption are important potential confounders in lung cancer risk assessment. Unfortunately, detailed smoking history data were not available in this retrospective study. Given that smoking is a major risk factor for lung cancer and may influence systemic inflammation markers, the absence of this variable represents an important limitation. Future studies should prospectively collect comprehensive smoking data to further validate our findings and adjust for this critical confounder.

Several limitations warrant consideration. First, the retrospective design and single-center recruitment may introduce selection bias. Second, while our model performed well in early-stage lung cancer, further validation in larger, multiethnic cohorts is needed to assess its applicability across diverse populations. Third, the inclusion of only treatment-naïve patients limits insights into biomarker dynamics during therapy. Future prospective studies should evaluate longitudinal changes in these markers and their predictive value for treatment response.

In conclusion, our study presents a robust, non-invasive diagnostic model combining ProGRP and inflammation-related biomarkers for lung cancer detection. This approach addresses key challenges in lung cancer screening, including cost-effectiveness and radiation exposure concerns associated with LDCT, given the rising global burden of lung cancer, such biomarker-based strategies could enhance early detection, particularly in resource-limited settings. Further integration with imaging and genomic data may refine risk prediction and personalize lung cancer management.

## Data Availability

The datasets and materials used and analyzed during the current study are available from the corresponding author on reasonable request.

## Abbreviations

CYFRA21-1: cytokeratin 19 fragment
NSE: Neuron-Specific Enolase
SCCA: Squamous Cell Carcinoma Antigen
ProGRP: Pro-Gastrin-Releasing Peptide
IL-1β: Interleukin-1 beta
IL-6: Interleukin-6
TNF-α: Tumor Necrosis Factor-alpha
IFN-γ: Interferon-gamma
IL-12p70: Interleukin-12p70
NE: Neutrophil Count
LY: Lymphocyte Count
MO: Monocyte Count
PLT: Platelet Count
SII: Systemic Immune-Inflammation Index
NLR: Neutrophil-to-Lymphocyte Ratio
PLR: Platelet-to-Lymphocyte Ratio
LMR: Lymphocyte-to-Monocyte Ratio
ROC: Receiver operating characteristic curve
DCA: Decision curve analysis
